# miR-30 Family: A Promising Regulator in Development and Disease

**DOI:** 10.1155/2018/9623412

**Published:** 2018-05-29

**Authors:** Ling Mao, Shiming Liu, Lin Hu, Li Jia, Hairong Wang, Mengmeng Guo, Chao Chen, Yun Liu, Lin Xu

**Affiliations:** ^1^Special Key Laboratory of Gene Detection & Therapy of Guizhou Province, Zunyi Medical College, Zunyi, Guizhou 563003, China; ^2^Guizhou Provincial College-Based Key Lab for Tumor Prevention and Treatment with Distinctive Medicines, Zunyi Medical College, Zunyi, Guizhou 563003, China

## Abstract

MicroRNAs (miRNAs) are small noncoding RNAs that negatively regulate posttranscriptional expression of target genes. Accumulating evidences have demonstrated that the miR-30 family, as a member of microRNAs, played a crucial regulating role in the development of tissues and organs and the pathogenesis of clinical diseases, which indicated that it may be a promising regulator in development and disease. This review aims to clarify the current progress on the regulating role of miR-30 family in tissues and organs development and related disease and highlight their research prospective in the future.

## 1. Introduction

MicroRNAs (miRNAs) are a family of 20~25-nucleotide small RNAs that generated from primary miRNA (pri-miRNA) transcribed by RNA polymerase II (Pol II). In the nucleus, the RNase III endonuclease Drosha and the double-stranded RNA-binding domain (dSRBD) protein DGCR8/Pasha cut one strand of the stem of the pri-miRNA hairpin and liberate a ~70-nucleotide stem-loop called a “pre-miRNA.” Exportin-5 transports the pre-miRNA into the cytoplasm and Dicer cuts pre-miRNA, together with the dSRBD protein TRBP/Loquacious, to generate the miRNA duplex. The miRNA strand is loaded into an Argonaute-containing RNA-induced silencing complex (RISC), which recognizes target mRNAs and commonly results in translational inhibition or destabilization of the target mRNA [1–3]. Up to now, miRNAs have been well documented as critical regulator in the development of tissues and organs and the pathogenesis of clinical diseases [4, 5].

**Figure 1:**
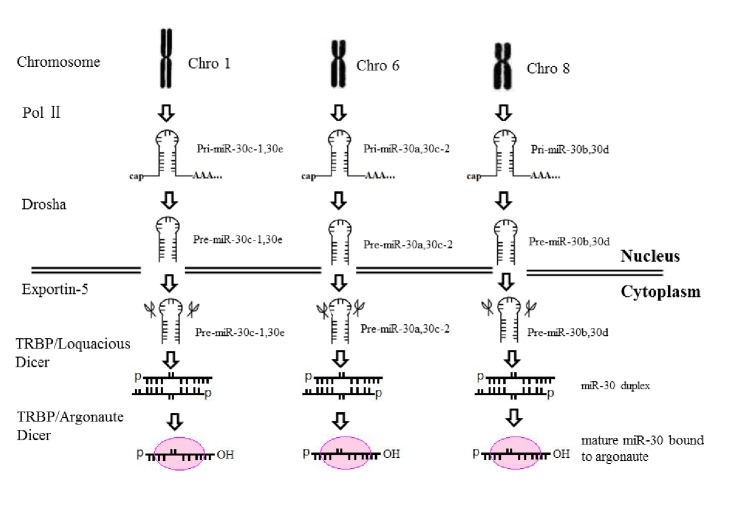
The biogenesis of miR-30 family.

The microRNA-30 (miR-30) family is an important member of miRNA family, which contains five members and 6 mature miRNA molecules (namely, miR-30a, miR-30b, miR-30c-1, miR-30c-2, miR-30d, and miR-30e) and is encoded by six genes located on human chromosome 1, 6, and 8 (Figure 1). These mature miRNAs share a common seed sequence near the 5′ end but possess different compensatory sequences near the 3′ end (Table 1). These different compensatory sequences allow miR-30 family members to target different genes and pathways, thus performing corresponding biological function [6]. Recent studies have shown that miRNA-30 family played an important regulatory role in tissues and organs development, as well as related clinical diseases pathogenesis.

## 2. miR-30 Family and Tissue and Organ Development

### 2.1. miR-30 Family and Bone Tissue

As early as in 2008, Li et al. reported that the expression of miR-30a and miR-30d decreased significantly in the differentiation of mesenchymal stem cell C2C12 induced by bone morphogenetic protein-2 (BMP-2) [7]. Subsequently, Wu et al. further discovered that miR-30 family members (including miR-30a, miR-30b, miR-30c, and miR-30d) could target transcription signaling molecule SMAD family member 1 (Smad1) and runt-related transcription factor 2 (Runx2) and negatively regulated BMP-2-induced differentiation of osteoblastic cells. However, the expression of miR-30e did not change in osteoblastic cells with BMP-2 stimulation, indicating that miR-30 family played an important role in osteoblast differentiation, but the exact roles of distinct miR-30 family members during osteogenic differentiation might be different [8]. In addition, Zhang et al. found that, with the stimulation of bone morphogenetic protein-9 (BMP-9), mesenchymal stem cell C3H10T1/2 could differentiate into osteoblastic cells. In this process, miR-30a played an important negative regulatory role. In detail, the expression of miR-30a in BMP-9-stimulated MSCs decreased at early stage and then increased at late stage of osteogenic differentiation. Moreover, miR-30a overexpression could affect the proliferation of MSCs, leading to a reduction in the expression of early osteogenesis marker Runx2 and late osteogenesis marker osteopontin. The mechanism may be related to the altered expression of Runx2, which was regulated by miR-30a [9]. Finally, other literatures also have documented that the expression of miR-30 family members, such as miR-30d and miR-30e, has also changed during the differentiation of MSCs into osteoblastic cells [30]. In conclusion, these studies suggested that miR-30 family participated in bone development. However, the exact role of miR-30 family and its mechanism in promoting embryonic bone formation remain to be elucidated in following studies.

### 2.2. miR-30 Family and Reproductive System

miR-30 was reported to be highly expressed in both mouse and human testes tissue and associated with the Homeobox protein and Zn transport, which were critical for male fertility [31], indicating that miR-30 played a role in reproductive development. Fischer et al. further showed that miR-30 family could regulate the generation of recombinant proteins in Chinese hamster ovary cells [32]. In terms of the definite mechanism, their latest research found that S-phase kinase related protein 2 (Skp2) and ubiquitin-conjugating enzyme E2, J1 (Ubej1) were regulated by miR-30 family in Chinese hamster ovary cells. Their data suggested that miR-30 family could affect the expression of recombinant proteins by regulating ubiquitin E3 ligase-Skp2-induced ubiquitin pathway, thereby affecting the development of reproductive system [10]. In addition, other researchers have found that miR-30 family was also robustly expressed in unfertilized rainbow trout eggs, which provided a new point to control the quality of eggs and early embryo formation of rainbow trout in the future [33]. The above studies showed that miR-30 family was involved in the animal reproductive system development, but their role in the development of human reproductive system and the underlying mechanism required further investigation.

### 2.3. miR-30 Family and Adipogenesis

Recent studies have found that miR-30 family played an important role in adipocyte differentiation. The expression of miR-30 family members vigorously increased in the differentiation of human adipose tissue-derived stem cells into adipocytes [34]. Moreover, the inhibition of miR-30 family members (including miR-30a and miR-30d) could reduce the process of lipogenesis. Conversely, the overexpression of miR-30 family members could promote the process of lipogenesis. The corresponding mechanism may be related to the expression of the transcription factor Runx2, which was a target of miR-30 family [11]. In addition, Hu et al. found that the expression of miR-30 family members (including miR-30b and miR-30c) was upregulated during adipose differentiation. Moreover, forced expression of miR-30b/c also significantly increased thermogenic gene expression in primary adipocytes. Mechanistic aspect, the knockdown of miR-30 family members (including miR-30b and miR-30c), could inhibit the expression of uncoupling protein 1 (UCP1) and cell death-inducing DFFA-like effector a (Cidea) in brown adipocytes, which was related to upregulation of their target RIP140 [12]. Finally, in an* in vivo *study, Li et al. found an increased expression of miR-30e in animal adipose tissue by using Solexa sequencing [35]. In summary, miR-30 family not only played an important role in adipogenesis but also participated in the regulation of brown adipose tissue function, indicating that it may be a new potential target for regulating lipid metabolism.

### 2.4. miR-30 Family and the Development of Other Normal Tissues and Organs

Multiple studies have suggested that miR-30 family members were involved in the development of other tissues and organs, including pancreas, blood vessels, and intestinal tissues, which showed the extensive roles of miR-30 family. For instance, Joglekar found that the concentration of miR-30 family was high in human islets and miR-30 family had participated in the intracellular response of pancreatic epithelial cells through regulating relative signaling pathways in epithelial mesenchymal transition (EMT) [36]. Bridge et al. found that miR-30 family members (including miR-30b and miR-30c) have taken part in the endothelial cell growth process by regulating the expression of *δ*-like ligand 4 (DLL4) during angiogenesis [13]. Moreover, Peck et al. reported that the inhibition of miR-30 family in intestinal epithelial cells resulted in a significant decrease in cell proliferation and a significant increase in the differentiation, indicating that miR-30 family had an important regulatory role in this differentiation process [37]. Finally, there were other studies indicating that miR-30b was also involved in mammary gland development and the expression of miR-30b was related to the lactation and involution [38]. Overall, these findings suggested that miR-30 family might take part in the development of multiple tissues and organs; however, the exact mechanisms need to be further elucidated.

## 3. miR-30 Family and Clinical Diseases

### 3.1. miR-30 Family and Cancer

#### 3.1.1. miR-30 Family as Tumor Suppressor miRNAs

Current studies have shown that miR-30 family, as tumor suppressor, played important roles in the development of various cancers. For example, Cheng et al. found that miR-30a could inhibit the metastasis and invasion of breast cancer cells by negatively regulating the expression of the vimentin [39]. And Braun et al. reported that miR-30 could reduce the invasive potential of mesenchymal anaplastic thyroid carcinoma-derived cells [40]. Consistently, Zhong et al. reported that the inhibition of miR-30c promoted the invasion of non-small cell lung cancer by promoting EMT process [41]. Furthermore, recent studies have found that miR-30 family could inhibit tumor cell growth, which was related to the change of tumor cell autophagy. For example, Singh et al. found that the restoration of miR-30a weakened the tumorigenesis of medulloblastoma cells, accompanied with a decreased expression in Beclin 1 and the inhibition of autophagy [14]. Moreover, Zhang et al. found that miR-30d could inhibit autophagy of colon cancer cells by directly targeting messenger RNA of autophagy related protein 5 (ATG5), Beclin 1, and phosphoinositide 3-kinase (PI3K), thereby promoting cell apoptosis [15].

#### 3.1.2. miR-30 Family as Oncogenic miRNAs

Similar to other miRNAs, such as miR-7 [42], miR-30 family also played a role, as oncogenic miRNA, in tumorigenesis of some cancers, which reflected the complexities of their biological function. For example, Wang et al. found that the overexpression of miR-30a promoted tumor formation by inhibiting the expression of forkhead box protein L2 (FOXL2) in COV434 cells, accompanied with the upregulation of B-cell lymphoma 2 related protein A1 (BCL2A1), immediate early response 3 (IER3), and cyclin D2 [16]. Moreover, the level of miR-30a was upregulated in the urine of ovarian serous adenocarcinoma patients [43]. Consistently, knockdown of miR-30a suppressed the malignant phenotypes of ovarian cancer cells* in vitro* [43]. Most interestingly, Gaziel-Sovran et al. further found that miR-30 family members (including miR-30b and miR-30d) promoted the metastatic behavior of melanoma cells by directly targeting the N-acetyl galactose (GalNAc) transferase GALNT7, resulting in increased synthesis of the immunosuppressive cytokine interleukin-10 (IL-10) and decreased immune cells activation and recruitment [17]. This research work suggested that miR-30 family could contribute to the development of cancer partially through affecting host immune reaction.

In summary, to the contradictory roles of miR-30 family in tumorigenesis, we infer that this may reflect the biological function of miR-30 family, which was related to the different tumor types, the expression level of miR-30 family, and corresponding various target molecules in distinct types of cancers. Therefore, it is necessary to further elucidate the definite function and its mechanism of miR-30 family in related cancers.

### 3.2. miR-30 Family and Cardiovascular Disease

Nakagawa et al. found that the expression of miR-30, which could target cardiac GalNAc-transferase (GALNT) 1 and 2 expression, was abundant in myocardium of healthy hearts but decreased obviously in failing hearts [44]. Furthermore, miR-30e mimic treatment resulted in downregulation of Beclin 1 via inhibiting its 3′UTR activity and protected primary cardiomyocytes against apoptosis* in vitro*. Conversely, miR-30e silencing promoted cardiomyocytes apoptosis by elevating the expression of angiotensin-converting enzyme 2 (ACE2), thereby reducing the apoptosis of cardiomyocytes [18]. However, it would be noticed that, in animal cardiac ischemic injury model, the silence of miR-30 family could elevate the levels of CSE and H_2_S and protect cardiomyocytes against hypoxia-induced injury* in vivo*, indicating the promising therapeutic potential of miR-30 family in ischemic heart diseases [45]. Thus, to this phenomenon, we presume that the controversial finding might be related to the difference on* in vitro* and* in vivo* experimental setting.

In addition, Zhang et al. found that high fat-induced miR-30 upregulation could impair the protective effects of endothelial cell autophagy against atherosclerosis through suppressing protein translation of ATG6 [19]. Finally, other studies showed that miR-30a could downregulate endothelial DLL4 expression, thereby controlling the behavior of tip cells, indicating that miR-30 family was also related to the rarefaction process and hypertension [20]. Hence, the investigation of miR-30 family is much helpful for the illustration on the pathogenesis and the development of novel therapeutic strategies of cardiovascular diseases.

### 3.3. miR-30 Family and Renal Disease

miR-30 family members are also involved in the pathological process of renal diseases [46]. For example, Wu et al. found that glucocorticoid-sustained miR-30 expression was associated with reduced Notch1 activation and alleviated podocytes damage, accompanied with altered expression of Notch1 and p53 [21], indicating the important role of miR-30 family in renal diseases. In line with this finding, Wu et al. further found that some critical components of calcium/calcineurin signaling, including TRPC6, PPP3CA, PPP3CB, PPP3R1, and NFATC3, were the targets of miR-30 family. Moreover, podocyte-specific expression of the miR-30 sponge in mice increased calcium/calcineurin pathway component protein expression and calcineurin activity [47]. Notably, recent evidence further showed that transforming growth factor-*β* (TGF-*β*) could inhibit the expression of miR-30d through a Smad2/3-HDAC3-NcoR repression complex and provide new insights into a potential target for the treatment of podocytes injury-associated glomerulopathies [22]. In addition, it has been reported that the levels of miR-30 family members (including miR-30a, miR-30c, and miR-30e) increased in the plasma of contrast-induced nephropathy (CIN) rats compared with their levels in non-CIN control rats, suggesting that miR-30 family might serve as early biomarkers and target candidates for CIN [48]. Moreover, miR-30c was also involved in the regulation of renal tubular cell apoptosis in cisplatin-induced nephrotoxicity, which provided a new therapeutic strategy for the improvement of cisplatin-induced nephrotoxicity [49].

Combining these studies suggested that miR-30 family played an important regulatory role in the pathogenesis of various kidney diseases and may be a potential target for treatment and diagnosis of relative diseases.

### 3.4. miR-30 Family and Other Clinical Diseases

Up to now, a large number of studies have demonstrated that miR-30 family has been involved in the pathogenesis of many other diseases, such as osteoarthritis, fibrosis, and hepatitis [50]. For example, Li et al. found that miR-30b was involved in the pathogenesis of osteoarthritis and its expression level in the articular cartilage increased robustly compared with normal people [23]. As for fibrosis diseases, Tu et al. reported that miR-30 could inhibit the occurrence of liver fibrosis, potentially through inhibiting its target molecule TGF-*β* [24]. Berschneider et al. further found that miR-30a could reverse WNT1-induced signaling pathway protein 1 (WISP1) mRNA expression in lung fibrosis, thereby affecting the process of lung fibrosis [25]. Importantly, miR-30 family was also reported to be involved in immune responses and related diseases. For example, miR-30a exhibited low expression in* Mycobacterium tuberculosis*- (MTB-) infected patients and the overexpression of miR-30 suppressed the ability of host cells to eradicate intracellular MTB [51]. Interestingly, the most recent evidence further showed that, in uninfected THP-1 cells, miR-30a could inhibit the expression of myeloid differentiation factor 88 (MyD88) and subsequently reduced the TLR signaling and cytokine expression, indicating that miR-30a participated in MTB-induced immune responses via regulating TLR/MyD88 activation and cytokine expression [26]. Besides, other studies have also found that miR-30 family was related to the pathogenesis of other diseases such as allergic rhinitis [52] and Alzheimer's disease [53]. However, the related mechanisms remain to be elucidated.

## 4. Conclusion

Current studies have shown that miR-30 family, as a member of miRNA family, played an important and promising role in the development of tissues and organs and various clinical diseases. Although existent investigations have revealed the related molecular mechanisms (Table 2), there are still many critical problems that need to be further illustrated in the future, such as (a) how to explore the exact mechanism of distinct miR-30 family members on regulating the development of different tissues and organs, (b) what are the exact roles of miR-30 family members in different types of tumors, (c) how to utilize the difference of miR-30 family members to perform subsequent targeted treatment, and (d) what are the exact roles of miR-30 family members in immune organs development and diseases-related immune responses. We believe that the elucidation of these representative questions not only will help to deepen the understanding of the biological function of miR-30 family but also will bring about a novel insight into diagnosis and molecular targeted therapy of related clinical diseases.

## Figures and Tables

**Table 1:** The sequences of miR-30 family members.

miRNAs	Pre-miRNA sequences	Mature miRNA sequences
miR-30a	5'-GCGACUGUAAACAUCCUCGACUGGAAGCUGUGAAGCCACAGAUGGGCUUUCAGUCGGAUGUUUGCAGCUGC-3'	Has-miR-30a-5p: UGUAAACAUCCUCGACUGGAAG
		Has-miR-30a-3p: CUUUCAGUCGGAUGUUUGCAGC
miR-30b	5'-ACCAAGUUUCAGUUCAUGUAAACAUCCUACACUCAGCUGUAAUACAUGGAUUGGCUGGGAGGUGGAUGUUUACUUCAGCUGACUUGGA-3'	Has-miR-30b-5p: UGUAAACAUCCUACACUCAGCU
		Has-miR-30b-3p: CUGGGAGGUGGAUGUUUACUUC
miR-30c-1	5'-ACCAUGCUGUAGUGUGUGUAAACAUCCUACACUCUCAGCUGUGAGCUCAAGGUGGCUGGGAGAGGGUUGUUUACUCCUUCUGCCAUGGA-3'	Has-miR-30c-1-5p: UGUAAACAUCCUACACUCUCAGC
		Has-miR-30c-1-3p: CUGGGAGAGGGUUGUUUACUCC
miR-30c-2	5'-AGAUACUGUAAACAUCCUACACUCUCAGCUGUGGAAAGUAAGAAAGCUGGGAGAAGGCUGUUUACUCUUUCU-3'	Has-miR-30c-2-5p: UGUAAACAUCCUACACUCUCAGC
		Has-miR-30c-2-3p: CUGGGAGAAGGCUGUUUACUCU
miR-30d	5'-GUUGUUGUAAACAUCCCCGACUGGAAGCUGUAAGACACAGCUAAGCUUUCAGUCAGAUGUUUGCUGCUAC-3'	Has-miR-30d-5p: UGUAAACAUCCCCGACUGGAAG
		Has-miR-30d-3p: CUUUCAGUCAGAUGUUUGCUGC
miR-30e	5'-GGGCAGUCUUUGCUACUGUAAACAUCCUUGACUGGAAGCUGUAAGGUGUUCAGAGGAGCUUUCAGUCGGAUGUUUACAGCGGCAGGCUGCCA-3'	Has-miR-30e-5p: UGUAAACAUCCUUGACUGGAAG
		Has-miR-30e-3p: CUUUCAGUCGGAUGUUUACAGC

**Table 2:** The targets of miR-30 family members in tissue and organ development and clinical diseases.

	Types	miRNAs	Targets	Cells/Models	References
Tissue and organ development	Bone tissue	miR-30a, miR-30d miR-30a, miR-30b, miR-30c, miR-30d miR-30a,	BMP2 Smad1, Runx2 Runx2	C2C12 cells MC3T3-E1 cells C3H10T1/2 cells	[7] [8] [9]
	Reproductive system	miR-30 family	Skp2, Ubej1	CHO cells	[10]
	Adipose tissue	miR-30a, miR-30d	Runx2	hMADS cells	[11]
		miR-30b, miR-30c	UCP1, Cidea, RIP140	C57BJ6 mice	[12]
	Vessel	miR-30b, miR-30c	DLL4	LECs, HUVECs	[13]

Related diseases	Tumorigenesis (as tumor suppressor)	miR-30a	Beclin1	Daoy, D283, D425 cells	[14]
		miR-30d	ATG5, PI3K, Beclin1	HCT15, HCT116, HT-29, DLD-1, SW480 cells	[15]
	Tumorigenesis (as oncogene)	miR-30a	FOXL2, BCLA1, IER3, cyclin D2	COV434 cells	[16]
		miR-30b, miR-30d	GALNT7	Melanoma cells, clinical specimens	[17]
	Cardiovascular disease	miR-30e	ACE2	Sprague Dawley rats	[18]
		miR-30	ATG6	ApoE^(-/-)^ mice, HAECs	[19]
		miR-30a	DLL4	Zebrafish	[20]
	Renal disease	miR-30 family	Notch1, p53	Wistar rats, human podocytes, clinical specimens	[21]
		miR-30d	TGF-*β*	HEK293 cells	[22]
	Osteoarthritis	miR-30b	ERG	SW1353 cells, clinical specimens	[23]
	Hepatic fibrosis	miR-30	TGF-*β*	ICR mice, HSCs	[24]
	Pulmonary fibrosis	miR-30a	WISP1	Sprague Dawley rats, primary human lung fibroblasts	[25]
	Tuberculosis	miR-30a	MyD88	THP-1 cells	[26]
	Radiation damage	miR-30c	REDD1	hFOB cells, CD34^+^ cells	[27]
		miR-30b, miR-30c	NF-*κ*B	CD2F1 mice, CD34^+^ cells	[28]
		miR-30b, miR-30c	Mcl-1	CD2F1 mice, CD34^+^ cells	[29]

*****Notes*. *BMP2*: bone morphogenetic protein-2; *Smad1*: SMAD family member 1; *Runx2*: runt-related transcription factor 2; *Skp2*: S-phase kinase related protein 2; *Ubej1*: ubiquitin-conjugating enzyme E2, J1; *UCP1*: uncoupling protein 1; *Cidea*: cell death-inducing DFFA-like effector a; *DLL4*: *δ*-like ligand 4; *ATG5*: autophagy related protein 5; *PI3K*: phosphoinositide 3-kinase; *FOXL2*: forkhead box protein L2; *BCLA1*: B-cell lymphoma 2 related protein A1; *IER3*: immediate early response 3; IL-10: interleukin-10; *ACE2*: angiotensin-converting enzyme 2; *ATG6*: autophagy related protein 6; *TGF-β*: transforming growth factor-*β*; *ERG*: EST-related genes; *WISP1*: WNT1-induced signaling pathway protein 1; *MyD88*: bone marrow differentiation factor 88; *REDD1*: DNA damage inducible transcript 4; *NF-KB*: nuclear factor kappa B; *Mcl-1*: BCL2 family apoptosis regulator.
